# Seven Letters with Replies

**Published:** 1877-04

**Authors:** 


NEWFIELD, N. Y., Dec. 27, 1876.

DR. UP DE GRAFF:

*Dear Sir:*—Enclosed you will find the card of an eye doctor, who pretends to have studied with you two years, and claims to stop at the Clinton House, in Ithaca, every three months. This card I obtained from a farmer who had lost the sight of his eye by the blow from a chip. Harris agreed to restore sight in five weeks, for four dollars cash, and a note of eleven dollars. The farmer, however, refused all offers. This shows the necessity of following your advice in regard to traveling doctors.

Yours, &c., S. D. C.

The above mentioned chap's card reads, Dr. Harris, "Optician Oculist," (whatever that may be), 223 Maiden Lane, New York. Like all traveling mountebanks—whether spectacle venders or oculists—who claim to hail from this Institution, he and they are impostors and swindlers. Never trust a traveling doctor, nor purchase spectacles from a perambulating optician.

---

PATERSON, N. J., Jan. 7, 1877.

DR. THAD S. UP DE GRAFF:

*Dear Sir:*—We have taken your journal several years and find something to interest us in every number. We *learn* something and pick up many hints about taking care of our health, and if we do not make them practical it is our own fault.

Allow me to make a criticism on the name "BISTOURY." That's all well enough for Medical gentlemen like yourself, but common people want a pocket dictionary to refer to, and don't always have it with them. Why not give a name that every one can understand and pronounce.

We take your journal this year in connection with the Galaxy and Harpers Weekly, so I suppose need not enclose the usual fifty-five cents.

Very truly, H. B. C.

P. S. If you wish you may publish the above, and we may learn the sentiments of other readers.

The word BISTOURY signifies "a surgical instrument for making incisions." It is the principal knife made use of by surgeons. The name is peculiarly appropriate to this journal for the reason that we are constantly making incisions in the practice and character of the various mountebanks, of the quack doctor variety, who are wandering about the country. Pronounce it Biss-tree.

WEEDSPORT, N. Y., Feb. 27, 1877.

DR. UP DE GRAFF:

I am getting to feel the need of aid for my eyes from the effect of age, no doubt, (48),—and having been recommended to try Eye Cups, I have taken the liberty to ask your opinion as to their practicability. I do not intend to try an experiment on my eyes if I know it.

Will you please reply.

Very truly yours, W. A. L.

We have given our opinion and warning relative to the use of eye-cups, *in extenso*, heretofore. We refer our correspondent to the BISTOURY of April, 1872, for a full and detailed account of the eye-cup swindle. We will simply add here, that the device should never be used. It is of no possible advantage to the eyes, and is capable of doing much harm. We have seen three cases of dislocation of the lens produced by its use, in which blindness was the ultimate consequence. The idea that an elastic ball—like the eye, can be changed in form by means of "suction," or atmospheric pressure, save for the time that the pressure is applied, is false, as can be speedily determined by applying the force upon any rubber ball.

---

JAMESTOWN, GA., Jan. 10, 1877.

EDITOR BISTOURY:

*Dear Sir:*—May I give your incomparable contributor, B. H. Pratt, a text? His articles are beyond price, and the good they do will live after him. If he will write to my text, I will see that it shall be copied by the press of the "Solid South," and with due acknowledgements.

Our country is infested with "Womb Doctors." In every city and town there is one or more, who on every occasion parade their specula, and whose female devotees visit every lady whose digestion is disturbed, or who is hypochondrical and persuades her that she has the symptoms of womb disease, and that no one but Dr. Speculum can do her any good.

Dr. S. may be recognized by the fact that he never visits any woman more than twice, before he discovers that she has "Womb Disease," and then once or twice a week for from twelve to sixty months he visits her, and each time he assures her that she is "almost well," but so long as her patience and husband's purse holds out, she never gets entirely well, and while robbing her husband, Dr. S. is inducing in her a case of speculophobia.

Had I the facile pen of Dr. Pratt, I could do justice to the subject, but feeling incompetent, I suggest the subject to him.

Yours truly, G. W. J., M. D.

The subject which the doctor broaches is one much needing ventilation. We should say, judging from Dr. J.'s brief note, that he is fully competent to do the subject justice. Let us hear from you, Doctor.

---

VALATIE, N. Y., Jan. 14, 1877.

DR. UP DE GRAFF:

*Dear Sir:*—Enclosed, please find fifty-five cents for another year's subscription to the BISTOURY. I have taken it for the last year and like it first rate. I have only one fault to find with it, that is, it does not come often enough. Why do you not make it a monthly. If all your subscribers like it as well as I, they will be willing to pay any advance that would be necessary in the price of the subscription.

Yours, truly, L. S.

We have received hundreds of letters asking us to make the BISTOURY a monthly. We would be glad to do so, did but our time permit. Had we nothing to do but edit this journal, we are just vain enough to declare that we could make it sprightly, entertaining and exceedingly useful. But will our patrons please remember that we have a large institution filled with surgical cases, who occupy *all* the time allotted for labor, and that the BISTOURY is edited after midnight when the editor should be in bed and at rest. Many times have we been almost persuaded to abandon our journal to other hands, but we love it so, we cannot bear to part with it. So we plod along, hoping for the time to come when the young people coming up under our care, may be prepared to share our professional labors with us. When that time comes, we will give you a monthly that will even rival our quarterly. Watch and wait.

---

MOUNTAIN VALLEY, Comache Co., Texas,
January 1st, 1877.

THAD S. UP DE GRAFF, M. D.:

*Doctor:*—I read your inestimable BISTOURY 1874 and '75, up to January No. '76. I was at that time a resident of Caldwell Co., Texas. Enclosed please find sixty cents, for which please send the BISTOURY to Blanket Creek P. O., Brown Co., Texas, (which is my nearest P. O.) We have a Dr.'s law here in Texas, which makes it necessary for any and all M. D.'s to go before a board of physicians appointed by the District Judge of each judicial district, for examination. No difference, if they, the M. D.'s, who may wish to practice physic, have diplomas, they must undergo an examination at the hands of this learned board, provided they have not been regularly engaged in the practice of physic for five successive years prior to January 1st, 1875, (of this favored few I am one.) But this is not what I wanted to divulge. I wish simply to say this. The ostensible object of the law is, at least, to prohibit charlatans and mountebanks from the practice of physic within the limits of our state. But I fear it will not do it. In my humble judgment, the better and surer plan would be for every practicing physician to subscribe for the BISTOURY, and show it to his neighbors, thereby inducing them to subscribe for it also, for I am of the opinion if there were but two or three readers of the BISTOURY in every neighborhood, there would be no need of legislative enactments to prevent the "sovereign people" being imposed upon by traveling doctors. Neither would those proprietary medicine vendors find fools enough to support their traffic. I have been enabled, by the BISTOURY, to convince several friends that those patent medicines are worse than worthless, and that traveling doctors are a swindle. Hence my opinion. Wishing you a long and happy life, with a renewal of my subscription, is the only new-year's present that I can at this time offer you.

Respectfully, G. C. D.

The Doctor's letter explains itself. Of course, we would be glad to see his suggestion carried out and the BISTOURY made to circulate in every neighborhood—or post office, as it now does in every state and territory of our land.

---

CANANDAIGUA, N. Y., Mar. 26, 1877.

DR. UP DE GRAFF:

I enclose the circular of a traveling optician, who professes to cure cataract and various other eye-troubles, with his spectacles. Now, how the deuce can *cataract* be cured with spectacles?

Just explain this little matter for us, will you not, in next BISTOURY?

W.

The circular referred to, is issued by Dr. H. Joel, oculist and optician, hailing from Mt. Morris, N. Y. We know nothing of the excellence of this Doctor's (?) lenses, but we do know that he, in his circular, exhibits the grossest ignorance of the construction and diseases of the eye. Here, again, our rule holds good:—*Never* trust a traveling spectacle vender or quack doctor.

